# Navigating the Immune Challenge in Glioblastoma: Exploring Immunotherapeutic Avenues for Overcoming Immune Suppression

**DOI:** 10.7759/cureus.46089

**Published:** 2023-09-27

**Authors:** Prateek Jain, Shambhavi Vashist, Binay K Panjiyar

**Affiliations:** 1 Internal Medicine, Maulana Azad Medical College, Delhi, IND; 2 Internal Medicine, NC Medical College and Hospital, Panipat, IND; 3 Medicine, Harvard Medical School, Boston, USA; 4 Internal Medicine, California Institute of Behavioral Neurosciences & Psychology, Fairfield, USA

**Keywords:** immunotherapy, therapeutic cancer vaccine, oncolytic viruses, immune-checkpoint inhibitors, car t cell therapy, glioblastoma therapy, grade iv glioblastoma, recurrent glioblastoma multiforme

## Abstract

Glioblastoma multiforme (GBM) is a primary brain tumor known for its short survival time, typically 14-18 months from diagnosis to fatality. Managing GBM poses significant challenges due to factors like the formidable blood-brain barrier, the immunosuppressive conditions within GBM, and the intricacies of surgical procedures. Currently, the typical treatment for GBM combines surgical procedures, radiation therapy, and chemotherapy using temozolomide. Unfortunately, this conventional approach has not proven effective in substantially extending the lives of GBM patients. Consequently, researchers are exploring alternative methods for GBM management. One promising avenue receiving attention in recent years is immunotherapy. This approach has successfully treated cancer types like non-small cell lung cancer and blood-related malignancies. Various immunotherapeutic strategies are currently under investigation for GBM treatment, including checkpoint inhibitors, vaccines, chimeric antigen receptor (CAR) T-cell therapy, and oncolytic viruses. A comprehensive review of 26 high-quality studies conducted over the past decade, involving thorough searches of databases such as PubMed and Google Scholar, has been conducted. The findings from this review suggest that while immunotherapeutic strategies show promise, they face significant limitations and challenges in practical application for GBM treatment. The study emphasizes the importance of combining diverse approaches, customizing treatments for individual patients, and ongoing research efforts to improve GBM patients' outlook.

## Introduction and background

Gliomas, which include astrocytoma, oligodendroglioma, mixed glioma, medulloblastoma, and ependymoma, are the most common primary tumors originating from glial cells or their precursors in the central nervous system (CNS) [1]. Glioblastoma, categorized as a WHO grade IV astrocytoma, is adults' most prevalent primary brain tumor. This group of tumors is notably diverse and presents considerable challenges in terms of treatment due to its aggressive nature [2]. Each year, around five to six cases per 100,000 individuals are diagnosed with primary malignant brain tumors, with approximately 80% falling under the category of malignant gliomas (MGs). Glioblastoma multiforme (GBM) comprises more than half of MG cases and is associated with high morbidity and mortality [3]. GBM can be divided into primary and secondary types. Primary GBM arises spontaneously without evidence of a less malignant precursor, whereas secondary GBM develops from initially low-grade diffuse astrocytoma (WHO grade II) or anaplastic astrocytoma (WHO grade III). Most GBMs (~90%) are primary [4]. Patients with primary GBM tend to be older (average age = 55 years) than those with secondary GBM (average age = 40 years). Secondary GBM is linked to a more favorable prognosis and extended overall survival than primary GBM [4]. The current standard treatment for gliomas involves maximal surgical resection followed by concurrent radiation and chemotherapy with temozolomide (TMZ) within 30 days of surgery [5]. Nevertheless, this conventional treatment cannot ensure complete healing or the absence of tumor recurrence. Despite implementing aggressive post-surgery therapeutic regimens, the median survival for GBM patients is merely one year [6]. Adding TMZ to GBM treatment protocols resulted in a modest extension of patient survival by two and half months on average, reaching 15 months after diagnosis [6]. GBM is a highly malignant tumor with a bleak prognosis for four primary reasons: 1) gliomas are characterized by high proliferation, invasiveness, and angiogenesis, making them biologically aggressive; 2) gliomas exhibit widespread invasion of normal brain tissue, making complete surgical resection nearly impossible; 3) gliomas display significant diversity within the tumor, and 4) gliomas are known to suppress the immune system in the tumor microenvironment, allowing the tumor to evade the immune response and grow [7]. Historically, the brain has been considered an "immune privileged" organ due to the presence of the blood-brain barrier (BBB) and the absence of a lymphatic system. However, this concept was challenged in 2015 with the discovery of functional lymphatic vessels in the meninges, providing a direct drainage pathway to the cervical lymph nodes. Consequently, the brain is now regarded as immunologically distinct rather than privileged [8]. Immune cells can enter the CNS from the bloodstream when the BBB is compromised, as observed in glioblastoma through imaging with contrast agents. This disruption results in an immune-rich microenvironment within GBM, comprising a mixture of resident and infiltrating innate and adaptive immune cells. However, these immune cells often have suppressive characteristics, including macrophages and T regulatory lymphocytes (Tregs), leading to what is referred to as a "cold tumor" profile [9]. Given this immunosuppressive environment, novel therapeutic opportunities are emerging to reinvigorate immune responses against tumors, potentially enhancing tumor clearance and improving patient survival rates [10]. These innovative immunotherapeutic strategies for GBM encompass various approaches, such as checkpoint inhibitors, the delivery of oncolytic viruses, the adoption of chimeric antigen receptor (CAR) T cells, tumor or peptide vaccines, and dendritic cell therapies [11]. 

## Review

Methodology

Search Strategy

A thorough literature search was done on Google Scholar and PubMed databases, including MEDLINE. The search was done using keywords such as "immunotherapy" and "Glioblastoma Multiforme" and combining them using Boolean operators such as "AND" and "OR." A Medical Subject Headings (MeSH) strategy was used to narrow the published articles. Table 1 summarizes the databases screened for the collection of articles and the search strategy used for the same.

**Table 1:** Databases used for collecting articles (along with search strategies and appropriate filters).

Type of databases	Keywords	Search strategy	Filter used	Number of records identified
PubMed	Immunotherapy, Glioblastoma Multiforme	("Immunotherapy"[Majr]) AND (( "Glioblastoma/drug therapy"[Majr] OR "Glioblastoma/therapy"[Majr] ))	Free full text, last ten years, English.	163
Google Scholar	Immunotherapy, Glioblastoma Multiforme	Immunotherapy and Glioblastoma Multiforme	Published between 2013 and 2023	51

Inclusion and Exclusion Criteria

Only free full-text written in English published within the last 10 years, from August 1, 2013, to August 31, 2023, were included. All study designs were taken into consideration for this review. Grey literature was not included.

Results

**Figure 1:**
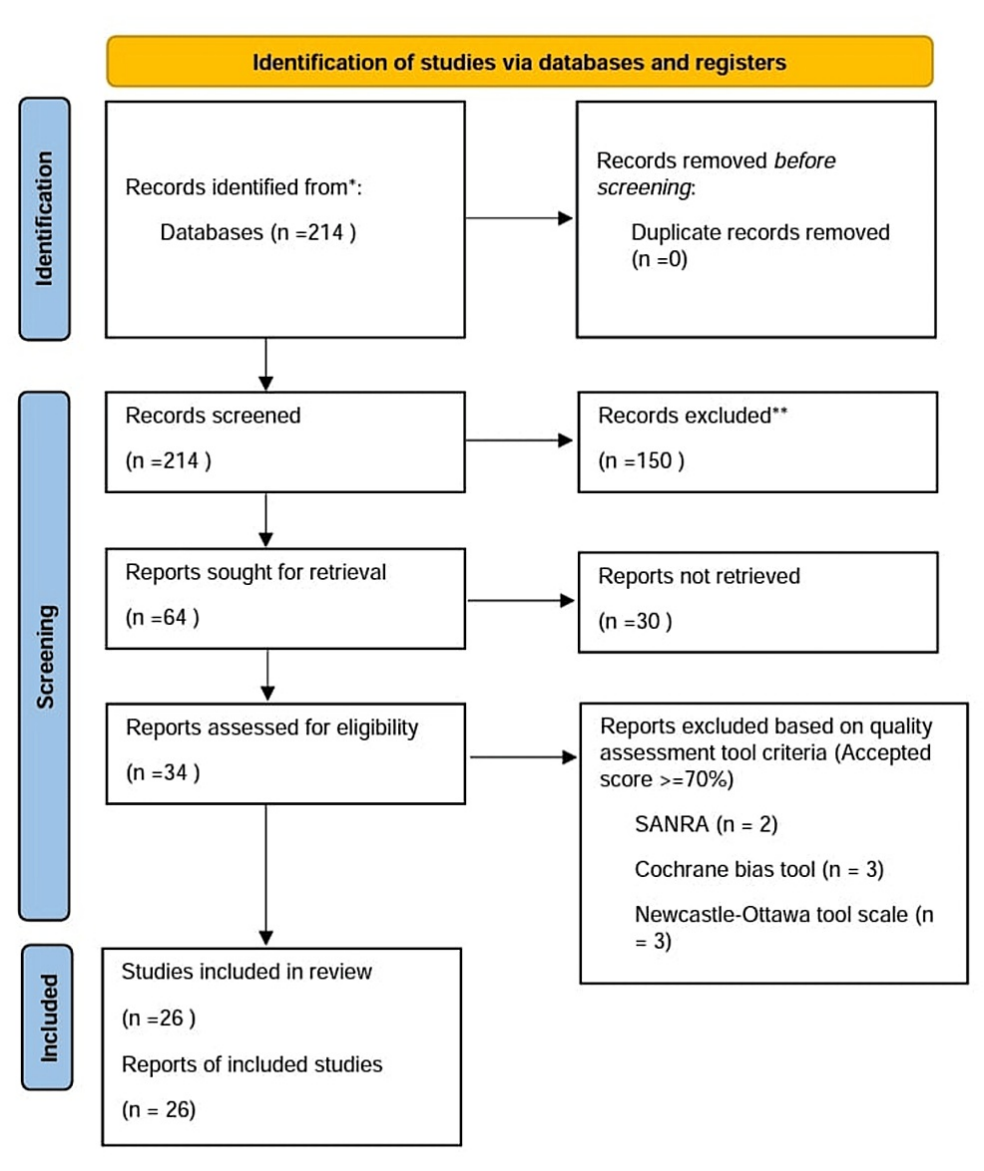
Preferred Reporting Items for Systematic Reviews and Meta-Analyses (PRISMA) chart illustrating the screening process and quality assessment of the articles. SANRA: Scale for the Quality Assessment of Narrative Review Articles; Cochrane Bias tool: Preferred for randomized controlled trials; Newcastle-Ottawa tool scale: Preferred for non-randomized clinical trials.

Using a search strategy and appropriate filters, 214 studies were identified from the databases, as mentioned above, within the last 10 years. Results were grouped in Excel (Microsoft, Redmond, WA, USA) to remove duplicates that were 0 in number. One hundred fifty were excluded based on irrelevant titles and abstracts. There were 64 studies left, which were thoroughly screened as full-text papers. Out of those, 30 were excluded. Quality assessment was then performed on the remaining 34 studies using quality assessment tools such as the Scale for the Assessment of Narrative Review Articles (SANRA) (for narrative review articles), Cochrane bias tool assessment for randomized clinical trials, and A Measurement Tool to Assess Systematic Reviews (AMSTAR 2) for systematic reviews and meta-analyses. Non-randomized clinical trials were evaluated using the Newcastle-Ottawa tool scale. A total of 26 studies that scored ≥70% on quality assessment were included in this review. An overview of the screening process is presented in the Preferred Reporting Items for Systematic Reviews and Meta-Analyses (PRISMA) chart, as shown in Figure 1. The studies included in this review are summarized in Table 2.

**Table 2:** Characteristics of studies included in this review. SANRA: Scale for the Assessment of Narrative Review Articles; AMSTAR: Assessment of Multiple Systematic Reviews; CAR: chimeric antigen receptor; PD-1: programmed cell death protein 1; PD-L1: programmed death-ligand 1; EGFRvIII: epidermal growth factor receptor variant III

Author/Year	Report Type	Quality assessment tool used	Database Used	Conclusion
Vázquez Cervantes et al., 2021 [10]	Review	SANRA	Google Scholar	The review discusses novel immunotherapeutic strategies for glioblastoma, highlighting their potential in improving treatment outcomes for this aggressive brain cancer.
Gedeon et al., 2020 [11]	Review	SANRA	PubMed	The review summarizes the current progress, challenges, and future prospects of checkpoint inhibitor immunotherapy for glioblastoma, highlighting the need for continued research and development in this promising but complex treatment approach.
Majc et al., 2021 [12]	Review	SANRA	Google Scholar	The review discusses current strategies and challenges in glioblastoma immunotherapy, emphasizing the importance of robust tumor model development for advancing effective treatments in this context.
Kamran et al., 2016 [13]	Review	SANRA	PubMed	This study discusses the recent advances and future prospects of immunotherapy for glioblastoma, emphasizing the need for further research and development.
Mende et al., 2021 [14]	Review	SANRA	PubMed	The study provides an overview of current advances in glioblastoma immunotherapy, highlighting promising approaches and their potential impact on patient outcomes.
Bausart et al., 2022 [15]	Review	SANRA	Google Scholar	This study explores combination strategies in immunotherapy for glioblastoma, underscoring their potential for improved treatment efficacy.
Huang et al., 2020 [16]	Review	SANRA	Google Scholar	The study summarizes current immunotherapies for glioblastoma, addressing the evolving landscape of treatments for this challenging cancer.
Daubon et al., 2020 [17]	Review	SANRA	PubMed	The study examines the immune landscape of glioblastoma and discusses the potential of new immunotherapies in addressing the disease.
Reardon et al., 2020 [18]	Clinical trial	Newcastle-Ottawa tool scale	PubMed	This clinical trial evaluates the effect of nivolumab vs. bevacizumab in recurrent glioblastoma, contributing valuable insights into immunotherapy outcomes.
Cloughesy et al., 2018 [19]	Randomised controlled trial	Cochrane bias tool	PubMed	The study demonstrates that neoadjuvant anti-PD-1 immunotherapy can promote a survival benefit in recurrent glioblastoma patients by eliciting intratumoral and systemic immune responses.
McGranahan et al., 2019 [20]	Review	SANRA	Google Scholar	The study provides an overview of the current state of immunotherapy for glioblastoma, discussing available treatment options.
Rocha Pinheiro et al., 2022 [21]	Review	SANRA	PubMed	This study offers insights into the current status and future prospects of immunotherapy in glioblastoma treatment.
Wu et al., 2021 [22]	Review	SANRA	PubMed	The study discusses the prospects of using antibodies targeting CD47 or CD24 in the treatment of glioblastoma.
Li et al., 2020 [23]	Review	SANRA	PubMed	The study explores chimeric antigen receptor T-cell therapy as a potential immunotherapeutic approach for glioblastoma.
Yuan et al., 2022 [24]	Review	SANRA	PubMed	This study reviews recent advances and future prospects in immunotherapy for glioblastoma.
Liau et al., 2022 [25]	Randomised controlled trial	Cochrane bias tool	PubMed	The study reports on a phase 3 trial involving autologous tumor lysate-loaded dendritic cell vaccination in glioblastoma patients, highlighting its potential for extending survival.
Das et al., 2023 [26]	Review	SANRA	Google Scholar	The study discusses mechanisms and clinical applications of immunotherapeutic approaches for glioblastoma multiforme.
Mahmoud et al., 2022 [27]	Review	SANRA	PubMed	This review explores recent advances in immunotherapy for glioblastoma multiforme
Reardon et al., 2016 [28]	Review	SANRA	PubMed	The study discusses advances in immunotherapy for glioblastoma, emphasizing the evolving landscape of treatment options.
Bagley et al., 2018 [29]	Review	SANRA	PubMed	The study discusses CAR T-cell therapy for glioblastoma, highlighting recent clinical advances and challenges.
O'Rourke et al., 2018 [30]	Clinical trial	Newcastle-Ottawa tool scale	PubMed	This study reports on the use of EGFRvIII-directed CAR T cells in glioblastoma patients and its impact on antigen loss and adaptive resistance.
Wang et al., 2019 [31]	Review	SANRA	PubMed	The study discusses challenges and potential in PD-1/PD-L1 checkpoint blockade immunotherapy for glioblastoma.
Zhang et al., 2022 [32]	Review	SANRA	Google Scholar	The study provides an overview of advances in immunotherapies for gliomas, including glioblastoma.
Hu et al., 2022 [33]	Systematic review and meta-analysis	AMSTAR-2	PubMed	This systematic review and meta-analysis examine the impact of molecular and clinical variables on survival outcomes with immunotherapy for glioblastoma patients.
Chowdhury et al., 2021 [34]	Review	SANRA	PubMed	The study discusses current advances in immunotherapy for glioblastoma multiforme and future prospects.
Khaddour et al., 2020 [35]	Review	SANRA	Google Scholar	The study reviews the landscape of novel therapeutics and challenges in glioblastoma multiforme, highlighting contemporary state and future directions.

Discussion

This section will focus on how glioblastoma multiforme creates an immunosuppressive microenvironment and different immunotherapeutic approaches currently employed in glioblastoma multiforme.

Mechanism of Immunosuppressive Microenvironment in GBM

Glioblastoma creates a complex immune-suppressed environment, affecting the overall body and the tumor site. In glioblastoma patients, standard treatments such as radiotherapy, temozolomide, and corticosteroids significantly reduce the strength of the immune responses, both adapting to new threats and acting instinctively [12]. The glioblastoma cells establish and maintain this immune-suppressive setting [13]. These cells produce interleukin (IL)-10 and transforming growth factor (TGF)-β that calm inflammation. These substances weaken the response of certain T cells and hinder the effectiveness of cells responsible for presenting antigens. In contrast, they boost the numbers and influence of regulatory T cells (Tregs). Additionally, glioblastoma cells release chemicals like CCL2 that attract Tregs to the tumor area [13]. Alongside this, the immune cell makeup in the glioblastoma's microenvironment is marked by an abundance of macrophages and specific myeloid cells, while lymphocytes are scarce. Tumor-associated macrophages (TAMs) contribute to glioblastoma's advancement by aiding the formation of new blood vessels and suppressing the body's adaptive immune response [14]. An integral part of maintaining the immune-suppressed environment within glioblastoma involves myeloid-derived suppressor cells (MDSCs), a diverse group of cells. These cells hinder immune responses by interacting with different cell types within the tumor environment. They bolster the function of regulatory T cells, hamper the display of antigens, and restrain the activity of certain T cells, among other functions. Importantly, myeloid cells like TAMs and MDSCs in glioblastoma patients have been found to produce more of a molecule called programmed death-ligand 1 (PD-L1), which negatively affects immune responses. This contributes to the disruption of T cell function and further curtails the body's natural defenses [15].

Different Immunotherapeutics Approaches for GBM

Checkpoint inhibitor immunotherapy: Inhibitory checkpoints are like built-in safeguards in our body that prevent the immune system from going into overdrive. Unfortunately, many cancers, including GBM, use these safeguards by increasing specific proteins that quiet down the immune response. One way to tackle this is by using checkpoint blockade, an approved method for treating solid tumors like melanoma and non-small-cell lung carcinoma (NSCLC) [11]. It focuses on vital immune checkpoints such as programmed cell death protein 1 (PD-1), PD-L1, and cytotoxic T-lymphocyte (CTL) antigen 4 (CTLA-4). We can block these checkpoints by using specialized antibodies, boosting the immune system. This has been successful in other cancers like melanoma and NSCLC [15]. 

In GBM, there has been progress, too. Blocking CTLA-4 and PD-1 together showed impressive results, even against more advanced tumors, in mice with GBM [16]. These approaches are being tested for newly diagnosed or recurrent GBM in clinical trials. However, the results for GBM have not been as promising as in other cancers, primarily when used alongside standard treatments [11].

For instance, a trial called CheckMate-143 compared nivolumab (anti-PD-1) and bevacizumab in recurrent GBM patients, but there was no significant difference in overall survival (OS) between the two treatments [11,17,18]. A similar trial, CheckMate-498, was stopped when nivolumab with radiation did not show better survival than temozolomide with radiation for newly diagnosed GBM patients without a specific gene alteration [14]. Another trial, CheckMate 548, evaluating nivolumab with or without radiation therapy and TMZ in O6-methylguanine-DNA methyltransferase (MGMT)-methylated recurrent GBM patients, also failed to show improved median OS [16].

However, a recent study by Cloughesy et al. demonstrated that recurrent glioblastoma patients receiving neoadjuvant treatment with pembrolizumab (anti-PD-1), followed by adjuvant therapy after surgery, had significantly improved OS compared to those receiving only adjuvant post-surgical pembrolizumab treatment [12,19]. In addition to CTLA-4 and PD-1/PD-L1 therapies, researchers are exploring other checkpoint targets like TIM-3 and CD39. TIM-3, present on various immune cells, can lead to T cell exhaustion similar to PD-1. CD39, an ectonucleotidase, has gained attention for hindering antitumor immunity [11,16]. A potential improvement might involve using checkpoint inhibitors alongside other treatments that enhance the immune system. It is crucial to know that checkpoint inhibitors can lead to significant side effects in the central nervous system. There is a genuine concern that an overly strong immune response in the brain could result in harmful effects [20].

To sum up, although checkpoint inhibitors show potential, we are still determining how they fit into treating GBM. There is a pressing need for further research to identify the most effective treatment combinations and to gain a deeper understanding of the genetic and immune aspects of GBM tumors. This knowledge will be crucial in using checkpoint inhibitors successfully down the line [16].

Vaccines: Researchers have developed and tested various vaccines to treat GBM by activating the immune system. These vaccines fall into the following categories, each with its unique immunological foundation [21]. The first category includes peptide and DNA vaccines. These vaccines use genetic information from the tumor, making them more specific and targeted. They tap into the tumor's characteristics [21]. The second category involves cellular vaccines, which use dendritic cells modified to carry tumor antigens. This approach aims to capitalize on the immune system's capabilities [21]. The third approach uses mRNA-based vaccines, often utilizing viral carriers. These vaccines employ genetic material to trigger an immune response [21]. All these strategies revolve around boosting the immune system's ability to respond to the tumor. The goal is to counteract the tumor's ability to evade the body's immune defenses [21]. 

Peptide vaccines: Peptide vaccines are composed of around eight to 30 amino acids. They are tailored to include tumor-specific antigens (TSA), arising from mutations found only in tumor cells, not in normal ones, or tumor-associated antigens (TAA), originating from normal proteins that are overly expressed in both tumor and normal tissues. Unlike other solid tumors, GBM has relatively few mutations, resulting in only a small portion used as TSA [16]. Currently, the focus of peptide vaccine investigations for GBM includes rindopepimut, IMA950, and isocitrate dehydrogenase 1 (IDH1) [16]. About 40% of glioblastomas overexpress epidermal growth factor receptor (EGFR), particularly EGFR variant III (EGFRvIII), which results from the loss of specific exons. This has led to rindopepimut (CDX-110), a synthetic 14-amino acid peptide combined with an immune-stimulating carrier protein called keyhole limpet hemocyanin (KLH) [22]. In a phase II clinical trial, 65 patients with EGFRvIII-positive GBM were treated with rindopepimut alongside standard adjuvant therapy. The results showed a progression-free survival (PFS) of 66% at five and a half months and a median OS of 21.8 months [23]. 

However, a phase III trial called ACT IV, evaluating rindopepimut in combination with standard treatment, was stopped due to a lack of substantial improvement in OS for newly diagnosed GBM patients. The variation in EGFRvIII expression within tumors or its loss leading to the growth of resistant cells could be factors contributing to this outcome [17]. 

Multi-peptide vaccines were developed to tackle the diversity of glioblastoma cells. These involve administering a combination of tumor-associated peptides overly expressed in glioblastoma cells. Unfortunately, this approach did not significantly enhance the OS of glioblastoma patients [12].

Another strategy, heat shock protein-peptide complexes 96 (HSPPC-96), aims to address this challenge. In the phase I clinical trial, HSPPC-96 vaccination triggered a tumor-specific immune response in most high-grade glioma patients. A subsequent phase II trial involving surgically resectable recurrent GBM patients treated with HSPPC-96-loaded antigens from patient-derived glioma tissue demonstrated promising results, with a median OS of 42.6 weeks and a six-month survival rate of 29.3%. These outcomes have prompted ongoing clinical trials [16].

Dendritic cell vaccines: Dendritic cells (DCs) are specialized antigen-presenting cells that activate various immune cells like CD4, CD8, natural killer (NK), and natural killer T (NKT) cells. The success of DC vaccination depends on factors such as how DCs are prepared, how they are loaded with tumor antigens, the way they are administered, and the use of adjuvants [13].

The strengths of dendritic cell-based therapies lie in their ability to trigger anti-tumour responses and increase the tumor's ability to provoke an immune response. They essentially bridge the gap between innate and adaptive immunity, which is especially important in cancers with weak immune responses, like glioblastoma [12]. 

A clinical trial involving 24 patients with recurrent malignant glioma explored DC therapy created with granulocyte-macrophage colony-stimulating factor and IL-4, with or without OK-432. These DCs were injected intradermally or both intratumorally and intradermally. The results showed that DC vaccines were well-tolerated with no adverse effects, and the patients with intratumoral and intradermal administrations had better survival than those with only intradermal administration. Additionally, T cell reactivity increased after DC vaccination [24]. 

A novel DC vaccine named DCVax-L has been developed using tumor lysate [16]. A phase III trial found that adding DCVax-L to standard treatment significantly improved OS for newly diagnosed and recurrent glioblastoma. The treatment was safe and showed promising long-term survival results [25]. Another DC-based vaccine called ICT-107 was designed for newly diagnosed GBM patients. While its impact on OS was insignificant, it showed therapeutic potential in specific patients [24]. Incorporating cytomegalovirus phosphoprotein 65 RNA (CMV pp65) into DC vaccines is another approach due to the presence of CMV in GBM. A phase I trial combining pp65-specific DCs with preconditioning showed promising PFS and OS rates [26].

CAR T-cell therapy: Tumor-associated antigens are molecules present in normal tissues but excessively expressed in cancerous cells. On the other hand, neoantigens are unique to tumors and result from genetic mutations in the tumor cell's DNA. Identifying these neoantigens and aberrantly expressed surface proteins in tumor cells has paved the way for innovative treatments like T lymphocytes armed with CARs and tumor-targeted vaccines, which enhance the immune system's targeted attack on tumors [10]. CAR T cells are engineered immune cells with synthetic antigen-specific receptors on their surface. They comprise three parts: a targeting part, usually a single-chain variable fragment (scFv), a transmembrane domain, and an intracellular signaling domain. When a CAR molecule binds to its target, it sparks an immune response tailored to that antigen. One remarkable feature of CAR T cells is their ability to target various antigens, bypassing the usual immune restrictions [27]. CAR T cells offer a solution to major histocompatibility complex (MHC) downregulation, a strategy used by cancers like GBM to evade the immune system. By combining antigen recognition and T cell activation, CAR T cells can mount robust immune responses against tumor-associated antigens often suppressed by the body's immune tolerance mechanisms [28]. CAR T-cell therapy involves introducing the CAR construct into a patient's T cells, which are then infused back into the patient. When the CAR encounters its target antigen, it triggers T cell activation, causing the release of cytokines, cytolytic effects, and T cell proliferation [29]. 

The technology has evolved from its early stages, with different generations of CARs incorporating various co-stimulatory and cytokine-expressing domains to enhance their effectiveness [22]. While CAR T therapy has shown incredible results in blood-related cancers, applying it to solid tumors like GBM has proven challenging [27]. Some promising avenues of research focus on CAR T cells designed to target specific molecules like human epidermal growth factor receptor 2 (HER2), EGFRvIII, and alpha receptor 2 of IL-13 (IL-13Rα2) associated with GBM. Researchers are also exploring different ways to administer this therapy locally or systematically [21].

EGFRvIII CAR T cells: EGFRvIII, which arises from the removal of exons two to seven, is the most prevalent variant of this receptor seen in human tumors. Approximately 40% of newly diagnosed GBMs exhibit an increase in the EGFR gene, and nearly half of these EGFR-amplified GBMs contain the constitutively active and cancer-causing EGFRvIII. The change in the amino acid sequence due to EGFRvIII leads to a unique glycine residue at the merging point of exons one and eight. This creates a distinct and immune-triggering site within EGFR's extracellular domain. Consequently, strategies involving vaccines and CAR T-cell therapies targeting EGFRvIII have been devised [29]. The initial clinical research exploring CAR T therapy targeting EGFRvIII was carried out by O'Rourke et al. [30]. They studied 10 patients with recurrent EGFRvIII-positive GBM. Their findings indicated that administering CAR T cells through infusion is a secure approach, as there were no signs of harmful effects outside the tumor's environment or cytokine release syndrome. Although the study's primary objective was not to assess therapy effectiveness, it was noted that GBM regression was not observed in any patient except for one patient who maintained stable disease for over 18 months [21,23,29].

However, further studies have indicated that EGFRvIII-CAR T cells have a limited impact on GBM. A 2019 clinical trial involving 18 GBM patients treated with anti-EGFRvIII CAR T cells reported a median PFS time of 1.3 months, except for one case lasting 12.5 months. Even though the amount of CAR cells administered could influence their persistence, substantial objective responses were seldom seen. This early-phase trial showed that anti-EGFRvIII CAR T cells did not yield significant clinical benefits in GBM patients [23]. Following this, subsequent trials focused on enhancing anti-EGFRvIII CAR T cells through modifications, like BiTE-EGFR CAR T cells, PDIA3 mutant EGFRvIII CAR T cells, and EGFR806-CAR T cells. These modifications aimed to enhance the effectiveness and safety of CAR T-cell treatment for GBM [23].

HER2 virus-specific CAR T cells: HER2 is a tumor-associated antigen present in around 80% of GBM cases. However, HER2 is not limited to tumor cells; it is also found in normal cells, which raises the potential for generating unintended immune reactions when targeted as an antigen [21]. In a recent phase I trial, 17 patients with progressive HER2-positive glioblastoma were treated with HER2-specific CAR-modified T cells. A second-generation CAR design was used to ensure safety, resulting in no reports of toxicities that could limit the dosage. Post-infusion, HER2-CAR T cells were detected in all patients, with varying peak levels over time. Although CAR T cells did not show significant expansion, they persisted for up to a year. The clinical outcomes demonstrated a partial response in one patient lasting more than nine months and stable disease in seven patients, with three remaining free from disease progression for 24 to 29 months [23,29]. This study leveraged virus-specific T cells expressing CARs, utilizing their dual function in targeting tumors and providing co-stimulation through native T cell receptors that engage latent virus antigens. CAR T cells specific to adenovirus, Epstein-Barr virus (EBV), and CMV were administered, which had previously been deemed safe in recipients of hematopoietic stem cell transplants. All patients exhibited CAR T cells with counterparts specific to viruses, particularly in CMV seropositive patients. Despite the modest expansion of CAR T cells, the trial showcased the safety and feasibility of introducing virus-specific CAR T cells to GBM through peripheral infusion, displaying promising indications of efficacy [29]. Another clinical study involving 10 consecutive GBM patients highlighted that HER2-specific T cells stimulated the proliferation of T cells and the secretion of interferon‐gamma (IFN‐γ) and IL-2 when interacting with HER2-positive autologous GBM cells [23].

IL-13Rα2 CAR T cells: IL-13Rα2 is another tumor-associated antigen found in up to 50% of GBMs. Despite its presence in normal tissue, it is notably absent in significant levels in normal brain tissue [21]. 

In 2004, researchers introduced an innovative approach to target GBM using IL-13Rα2-specific CAR T cells. They genetically modified these cells to express a membrane-tethered IL-13 cytokine chimeric T-cell antigen receptor, also known as zetakine. The adoptive transfer of IL-13-zetakine(+) CD8(+) CTL clones led to the regression of human GBM orthotopic xenografts in vivo [23]. IL-13Rα2-specific CAR T cells have been well-tolerated in clinical settings and structurally optimized to prevent unintended Fc interactions [22]. In a groundbreaking trial, a patient with recurrent GBM received direct intratumoral CAR T-cell infusions at a distant tumor recurrence site. This study showcased the booming manufacturing and delivery of IL-13Rα2-targeted CAR T cells using an implanted reservoir/catheter system. The treatment was well-tolerated, with manageable adverse events like headaches and temporary neurological deficits. Encouragingly, early indicators of anti-glioma activity were observed, including a rapid increase in necrotic tumor volume on MRI, a notable reduction in IL-13Rα2 tumor cell expression, and a promising extension of OS [29]. 

Oncolytic viruses: Recently, oncolytic viruses have gained significance as a strategy for treating various solid tumors. This approach has garnered attention due to these viruses' ability to selectively infect and replicate in tumor cells. Their therapeutic potential lies not only in reducing tumor size but also in reactivating antitumor immune responses. The effectiveness of oncolytic viruses against tumors hinges on their capacity to specifically infect and eliminate tumor cells and trigger both innate and adaptive antitumor immune responses [10]. The process begins with virus infection of tumor cells, which attracts the innate immune system, leading to the release of cytokines and the lysis of tumor cells. This, in turn, stimulates the generation of an adaptive immune response targeting new tumor antigens, potentially resulting in long-lasting immunotherapeutic effects [20]. Various oncolytic viruses, including retroviruses, adenoviruses, herpes simplex virus (HSV), polioviruses, and measles viruses, are currently being explored in these approaches. Some viruses are armed with immunoregulatory molecules like IL-12 and OX40 ligand, further enhancing their ability to activate innate and adaptive antitumor immune responses [12]. Adenoviruses, for instance, can be engineered to induce cell death, trigger an immunogenic antitumor response, or carry therapeutic genes [14].

An example is DNX-2401, a replication-competent adenovirus designed for selective replication in tumor cells with specific signaling characteristics [16]. Clinical trials involving DNX-2401 demonstrated tumor reduction and extended survival among glioblastoma patients [27]. Another strategy involves replication-deficient adenoviruses, like aglatimagene besadenovec, used as vectors to deliver tumoricidal genes, such as the HSV thymidine kinase. These genes convert a non-toxic compound, ganciclovir, into a toxic nucleotide analog, selectively affecting infected dividing cells. Phase II trials of this approach showed improved PFS and OS in GBM patients [17]. Another notable advancement is developing a live attenuated poliovirus vaccine, PVS-RIPO, engineered with a rhinovirus ribosome entry site for safety. This vaccine targets CD155 expressed on both tumor cells and antigen-presenting cells. Clinical trials have revealed promising outcomes, including increased OS rates and complete responses in certain patients [16]. A unique retrovirus that does not cause cell lysis has been used in clinical trials. It carries a gene called cytosine deaminase and works with the prodrug 5-fluorocytosine. When this virus infects tumor cells, the cytosine deaminase enzyme converts 5-fluorocytosine into a potent anti-cancer drug called 5-fluorouracil. Interestingly, this enzyme is not naturally found in human cells. This combined viral therapy has shown promising results in phase I trials for both primary and recurrent high-grade gliomas. It extended patients' survival, increased the immune response within the tumor microenvironment, and activated the body's immune defense [12]. While the initial phase I results were encouraging, the subsequent phase II/III trial did not improve OS compared to the standard of care group. This led to the trial ending prematurely. Other oncolytic viruses, like ParvOryx (an oncolytic H-1 parvovirus), Toca 511 (a retroviral replication-competent vector), Reovirus, and HSV type 1, have also shown promise in phase I/II trials for GBM patients [31]. In a more recent development, a Zika virus-based oncolytic virus demonstrated the ability to target specifically GBM stem cells (GSCs), which are the cells responsible for tumor growth. This suggests a potentially superior approach for brain tumor therapy. Moreover, combining three elements - anti-CTLA-4, anti-PD-1, and a recombinant oncolytic herpes simplex virus expressing mouse IL-12 (G47Δ-mIL-12) - resulted in curing most mice in two glioma models. This success emphasized the importance of combining different approaches in upcoming trials [17]. Currently, the focus is on combining oncolytic viruses with other immunotherapies, like immune checkpoint inhibitors and adoptive cell therapy. This combined approach aims to extend the positive clinical responses initiated by oncolytic viruses [12].

Combinatorial approaches: To achieve the best patient outcomes, immune-based therapies must create strong, specific, and lasting immune responses against tumors. This challenge demands approaches to counteract local and systemic factors suppressing the immune system. Thus, combining different strategies may be necessary [28]. Although single immune-based treatments have shown promise, they have not been successful enough in extending the survival of glioblastoma patients. Therefore, researchers are now looking into combined approaches that can work together to enhance the effectiveness of immune-based therapies [12]. One method involves combining immune checkpoint inhibitors (ICBs) with chemotherapy, particularly TMZ, a standard glioblastoma treatment. This combination aims to improve tumor recognition, eliminate cancer cells, and counteract immunosuppression while utilizing TMZ's potential to initiate an antitumor immune response. However, standard TMZ doses can lead to immune system suppression and limited benefits from anti-PD-1 therapies. Researchers are exploring modified doses or administration methods to enhance the effectiveness of ICBs. Using metronomic TMZ doses could prevent T cell exhaustion and maintain the benefits of anti-PD-1 therapies. Additionally, local administration of chemotherapy through wafers loaded with TMZ or carmustine could amplify anti-PD-1 effects by potentially increasing antigen presentation during chemotherapy-induced tumor cell death. This is supported by higher levels of dendritic cells, critical immune cells involved in antigen presentation [15]. Combining different immunotherapies that work together effectively is a promising approach to cancer treatment. In neuro-oncology, opportunities include combining IL-2 blockade with EGFRvIII and CMV antigen vaccination with T cell transfer. Clinical trials are exploring these combinations to assess their potential benefits [28]. Another approach involves combining therapies that target tumor survival and promote cell death, such as vascular endothelial growth factor (VEGF) inhibitors and gene therapy. In the GLOBE trial, the VEGF inhibitor bevacizumab was combined with the VB-111 adenovirus, which activates a pathway leading to cell death in tumor endothelial cells. While this combination showed promise in early phases, it did not extend OS compared to bevacizumab alone. The timing of administering VB-111 alongside bevacizumab might have influenced these outcomes [14]. Combining immunotherapies has become an essential aspect of checkpoint blockade strategies, with combinations like anti-CTLA-4 and anti-PD-1 significantly enhancing responses in certain cancers. Although combination therapies can lead to increased side effects, they also show increased efficacy by targeting multiple pathways suppressing the immune system. In preclinical models of glioblastoma, studies have demonstrated positive outcomes from combining checkpoint blockade targeting pathways like TIM-3, LAG-3, and IDO-1. This suggests that combination approaches hold promise for heterogeneous tumors like glioblastoma [32]. 

Limitations and Future Prospects of Glioblastoma Multiforme Management

GBMs represent a significant challenge in cancer treatment, with a limited prognosis despite advanced medical care, averaging 12 to 18 months of survival post-diagnosis. Several factors contribute to this grim outlook, including late-stage diagnosis, the difficulty of delivering chemotherapy due to the blood-brain barrier, a lack of immune cells in the brain, and GBMs' ability to suppress the immune system [13]. A key obstacle in translating potential treatments from lab studies to human patients is the difference between mouse models and human GBMs, as mice typically have uniform tumor characteristics, while human tumors vary greatly [32].

Recently, novel immunotherapies have emerged as potential treatments for glioblastoma. These include using antibodies against CD47 or CD24 and combining IL-6 and CD40 or IL-12 with CAR T immunotherapy [33]. Researchers are also searching for new molecular targets by studying the genetic makeup of GBM tissue. Immunotherapies and vaccines targeting neoantigens (unique tumor-related molecules) are in development to combat drug resistance. Additionally, trials are exploring treatments like histone-modifying enzymes and compounds like ACT001 to inhibit the AEBP1 signaling pathway for GBM [34].

Understanding the immune environment within glioblastoma and the interactions between intracranial and extracranial immune factors is crucial, as there is a focus on harnessing the immune system against GBM. Early use of immunotherapy in the neo-adjuvant setting shows promise but requires confirmation through comprehensive prospective randomized trials [35]. Given the complexity of GBM, effective treatment will likely involve a combination of approaches, including surgery, radiation, chemotherapy, and various immunotherapies to target different aspects of the disease [34].

Limitations

This study has a few limitations that should be acknowledged. Firstly, the coverage of literature was limited to selected databases, namely PubMed databases, including MEDLINE and Google Scholar. Consequently, only free full-text articles and studies published within the last 10 years were included, which may exclude potentially relevant information. The review only included papers published in English, excluding studies published in other languages. Moreover, there was a scarcity of studies investigating the role of immunotherapy in glioblastoma multiforme. 

## Conclusions

GBM presents a significant challenge in cancer treatment due to its aggressive nature and the complex immunosuppressive environment it creates. This review delved into the mechanisms behind GBM's ability to evade the immune system and explored various immunotherapeutic approaches. GBM establishes an intricate immunosuppressive microenvironment through factors like immune-inhibitory cytokines, recruitment of regulatory immune cells, and the dominance of certain immune cells within the tumor site. While promising, immunotherapies like checkpoint inhibitors, vaccines, CAR T-cell therapy, and oncolytic viruses have shown mixed results in clinical trials, with limited improvements in overall survival for GBM patients. To combat GBM effectively, researchers are investigating combinations of these approaches and identifying new molecular targets. Despite challenges, ongoing research strives to enhance the prognosis and quality of life for GBM patients by harnessing the power of immunotherapy and innovative strategies in the fight against this formidable brain cancer. Moving forward, further avenues for investigation in immunotherapy for GBM involve examining customized treatment methods specific to each patient's characteristics. Furthermore, it is crucial to make progress in comprehending the tumor microenvironment and creating treatments that counteract its ability to suppress the immune system. To achieve these goals, multidisciplinary teams must collaborate and work together, unraveling the complexities of GBM immunology and translating their findings into more effective clinical interventions. By doing so, we can transform the current treatment landscape and provide new hope and better outcomes for patients with this challenging brain cancer.

## References

[1] Wang J, Shen F, Yao Y, Wang LL, Zhu Y, Hu J (2020). Adoptive cell therapy: a novel and potential immunotherapy for glioblastoma. Front Oncol.

[2] Touat M, Idbaih A, Sanson M, Ligon KL (2017). Glioblastoma targeted therapy: updated approaches from recent biological insights. Ann Oncol.

[3] Alifieris C, Trafalis DT (2015). Glioblastoma multiforme: pathogenesis and treatment. Pharmacol Ther.

[4] Wilson TA, Karajannis MA, Harter DH (2014). Glioblastoma multiforme: state of the art and future therapeutics. Surg Neurol Int.

[5] Xu S, Tang L, Li X, Fan F, Liu Z (2020). Immunotherapy for glioma: current management and future application. Cancer Lett.

[6] Chistiakov DA, Chekhonin IV, Chekhonin VP (2017). The EGFR variant III mutant as a target for immunotherapy of glioblastoma multiforme. Eur J Pharmacol.

[7] Almeida ND, Klein AL, Hogan EA (2019). Cold atmospheric plasma as an adjunct to immunotherapy for glioblastoma multiforme. World Neurosurg.

[8] Hernández A, Domènech M, Muñoz-Mármol AM, Carrato C, Balana C (2021). Glioblastoma: relationship between metabolism and immunosuppressive microenvironment. Cells.

[9] Desland FA, Hormigo A (2020). The CNS and the brain tumor microenvironment: implications for glioblastoma immunotherapy. Int J Mol Sci.

[10] Vázquez Cervantes GI, González Esquivel DF, Gómez-Manzo S, Pineda B, Pérez de la Cruz V (2021). New immunotherapeutic approaches for glioblastoma. J Immunol Res.

[11] Gedeon PC, Champion CD, Rhodin KE (2020). Checkpoint inhibitor immunotherapy for glioblastoma: current progress, challenges and future outlook. Expert Rev Clin Pharmacol.

[12] Majc B, Novak M, Kopitar-Jerala N, Jewett A, Breznik B (2021). Immunotherapy of glioblastoma: current strategies and challenges in tumor model development. Cells.

[13] Kamran N, Calinescu A, Candolfi M (2016). Recent advances and future of immunotherapy for glioblastoma. Expert Opin Biol Ther.

[14] Mende AL, Schulte JD, Okada H, Clarke JL (2021). Current advances in immunotherapy for glioblastoma. Curr Oncol Rep.

[15] Bausart M, Préat V, Malfanti A (2022). Immunotherapy for glioblastoma: the promise of combination strategies. J Exp Clin Cancer Res.

[16] Huang B, Li X, Li Y, Zhang J, Zong Z, Zhang H (2020). Current immunotherapies for glioblastoma multiforme. Front Immunol.

[17] Daubon T, Hemadou A, Romero Garmendia I, Saleh M (2020). Glioblastoma immune landscape and the potential of new immunotherapies. Front Immunol.

[18] Reardon DA, Brandes AA, Omuro A (2020). Effect of nivolumab vs bevacizumab in patients with recurrent glioblastoma: the CheckMate 143 Phase 3 randomized clinical trial. JAMA Oncol.

[19] Cloughesy TF, Mochizuki AY, Orpilla JR (2019). Neoadjuvant anti-PD-1 immunotherapy promotes a survival benefit with intratumoral and systemic immune responses in recurrent glioblastoma. Nat Med.

[20] McGranahan T, Therkelsen KE, Ahmad S, Nagpal S (2019). Current state of immunotherapy for treatment of glioblastoma. Curr Treat Options Oncol.

[21] Rocha Pinheiro SL, Lemos FF, Marques HS (2023). Immunotherapy in glioblastoma treatment: current state and future prospects. World J Clin Oncol.

[22] Wu H, Liu J, Wang Z, Yuan W, Chen L (2021). Prospects of antibodies targeting CD47 or CD24 in the treatment of glioblastoma. CNS Neurosci Ther.

[23] Li L, Zhu X, Qian Y (2020). Chimeric antigen receptor T-cell therapy in glioblastoma: current and future. Front Immunol.

[24] Yuan B, Wang G, Tang X, Tong A, Zhou L (2022). Immunotherapy of glioblastoma: recent advances and future prospects. Hum Vaccin Immunother.

[25] Liau LM, Ashkan K, Brem S (2023). Association of autologous tumor lysate-loaded dendritic cell vaccination with extension of survival among patients with newly diagnosed and recurrent glioblastoma: a phase 3 prospective externally controlled cohort trial. JAMA Oncol.

[26] Das S, Dash BS, Premji TP, Chen JP (2023). Immunotherapeutic approaches for the treatment of glioblastoma multiforme: mechanism and clinical applications. Int J Mol Sci.

[27] Mahmoud AB, Ajina R, Aref S (2022). Advances in immunotherapy for glioblastoma multiforme. Front Immunol.

[28] Reardon DA, Freeman G, Wu C (2014). Immunotherapy advances for glioblastoma. Neuro Oncol.

[29] Bagley SJ, Desai AS, Linette GP, June CH, O'Rourke DM (2018). CAR T-cell therapy for glioblastoma: recent clinical advances and future challenges. Neuro Oncol.

[30] O'Rourke DM, Nasrallah MP, Desai A (2017). A single dose of peripherally infused EGFRvIII-directed CAR T cells mediates antigen loss and induces adaptive resistance in patients with recurrent glioblastoma. Sci Transl Med.

[31] Wang X, Guo G, Guan H, Yu Y, Lu J, Yu J (2019). Challenges and potential of PD-1/PD-L1 checkpoint blockade immunotherapy for glioblastoma. J Exp Clin Cancer Res.

[32] Zhang M, Choi J, Lim M (2022). Advances in immunotherapies for gliomas. Curr Neurol Neurosci Rep.

[33] Hu W, Liu H, Li Z, Liu J, Chen L (2022). Impact of molecular and clinical variables on survival outcome with immunotherapy for glioblastoma patients: a systematic review and meta-analysis. CNS Neurosci Ther.

[34] Chowdhury S, Bappy MH, Clocchiatti-Tuozzo S, Cheeti S, Chowdhury S, Patel V (2021). Current advances in immunotherapy for glioblastoma multiforme and future prospects. Cureus.

[35] Khaddour K, Johanns TM, Ansstas G (2020). The landscape of novel therapeutics and challenges in glioblastoma multiforme: contemporary state and future directions. Pharmaceuticals (Basel).

